# Assessment of the Spatial QRS-T Angle by Vectorcardiography: Current Data and Perspectives

**DOI:** 10.2174/157340309789317850

**Published:** 2009-11

**Authors:** Christina Voulgari, Nicholas Tentolouris

**Affiliations:** 1st Department of Propaedeutic Medicine, Athens University Medical School, Laiko General Hospital Athens, Greece

**Keywords:** Vectorcardiography, spatial ventricular gradient, spatial QRS-T angle, action potential duration, arrhythmogenicity.

## Abstract

The concept of the ventricular gradient (VG) was conceived in the 1930s and its calculation yielded information that was not otherwise obtainable. The VG was not utilized by clinicians at large because it was not easy to understand and its computation time-consuming. Spatial vectorcardiography is based on the concept of the VG. Its current major clinical use is to identify primary [heterogeneity of ventricular action potential (VAP) morphology] in the presence of secondary [heterogeneity in ventricular depolarization instants] T-wave abnormalities in an ECG. Nowadays, the calculation of the spatial VG can be computed on the basis of a regular routine ECG and contributes to localization of arrhythmogenic areas in the heart by assessing overall and local VAP duration heterogeneity. Recent population-based studies suggest that the spatial VG is a dominant ECG predictor of future cardiovascular events and death and it is superior to more conventional ECG parameters. Its assessment warrants consideration for intensified primary and secondary prevention efforts and can be included in everyday clinical practice. This review addresses the nature and diagnostic potential of the spatial VG. The main focus is the role of the spatial VG in ECG assessment of dispersion of repolarization, a key factor in arrhythmogeneity.

## INTRODUCTION

Wilson in 1934 wrote a classic communication in order to describe a method of analyzing the electrocardiogram (ECG) which has not been employed heretofore and which he believed yielded information not obtainable in other ways [1]. Wilson’s concept was called the ventricular gradient and was understood by only a few researchers and even fewer clinicians [2]. Many others wrote masterful articles on the subject, but their well-written articles produced no ground swell of interest in the ventricular gradient [3, 4]. They all failed to stimulate clinicians to use the ventricular gradient in their daily work and the determination of the ventricular gradient was characterised as not practical for general use in electrocardiography [5]. 

Vectorcardiography, based on Wilson’s concepts, was developed in the early 1950s and made it possible to use vector concepts in the routine analysis of tracings [6]. The spatial ventricular gradient, as obtained by vectorcardiography, contributed considerably to a better understanding of the ECG manifestations of the cardiac repolarization process. As already stated, its power lays in the ability to assess the primary factors that contribute to the T-wave (i.e., heterogeneity of action potential morphology throughout the ventricles) in the presence of secondary factors contributing to the T-wave (i.e., heterogeneity in ventricular depolarization instants). Where T-wave’s morphology is an ECG expression of heterogeneity of repolarization, the spatial ventricular gradient discriminates between primary or secondary causes of such heterogeneity. Besides, the spatial ventricular gradient body surface mapping of local components has emerged as a technique for assessing local ventricular action potential duration heterogeneity [6]. The latter is believed to contribute to localization of arrhythmogenic areas in the heart. The spatial ventricular gradient, which can be computed on the basis of a regular routine ECG and does not require body surface mapping, aims to assess the overall heterogeneity of ventricular action potential morphology [6]. The purpose of this review is to define and explain the spatial ventricular gradient and to discuss when its calculation is of clinical value today. Moreover, it addresses the nature and diagnostic potential of the spatial ventricular gradient and focuses in its role in ECG assessment of dispersion of repolarization, a key factor in arrhythmogeneity. 

## DEFINITION OF THE SPATIAL VENTRICULAR GRADIENT

A cell is considered to be depolarized when it loses all of its electrical charges; thus, it is said to be excited. This is followed by a period of electrical inactivity, which is followed by repolarization, i.e. the restoration of electrical charges. When all parts of the cell are able to restore the electrical charges after the same amount of time has elapsed, there is no time gradient. On the other hand, when a part of the cell restores its electrical charges following a delay in time which is greater than the time required for the rest of the cell to restore its electrical charges, a time gradient is produced. This is called a T-altering force or a T-delaying force. In other words, the ventricular gradient is the mathematical symbol that illustrates the variation of the duration of the excited state of a cell, or a group of cells (including the entire ventricular muscle) [6]. The spatial ventricular gradient represents the vectorial approach of the ventricular gradient and yields one single vector of given size and direction for one heartbeat of a given person. The more a given lead is sensitive for the electrical activity in a particular area of the heart, the more the spatial ventricular gradient in such a lead expresses the local properties of that part of the heart. The lead dependent approach of the spatial ventricular gradient in body surface mapping has proven to be useful for finding localized inhomogeneities in the heart [7]. 

In 1983 it was theoretically proven that the QRST integral is determined by spatial heterogeneity in the area under the action potential rather than by heterogeneity in action potential duration (APD) alone. Hence, heterogeneity in action potential resting amplitudes, peak amplitudes, upslopes, downslopes and durations all contribute to the spatial ventricular gradient. This somewhat more generalized concept casts the spatial ventricular gradient into an index of heterogeneity of action potential morphology in the ventricles of the heart [8]. 

## CALCULATION OF THE SPATIAL VENTRICULAR GRADIENT

**Fig. (1):**
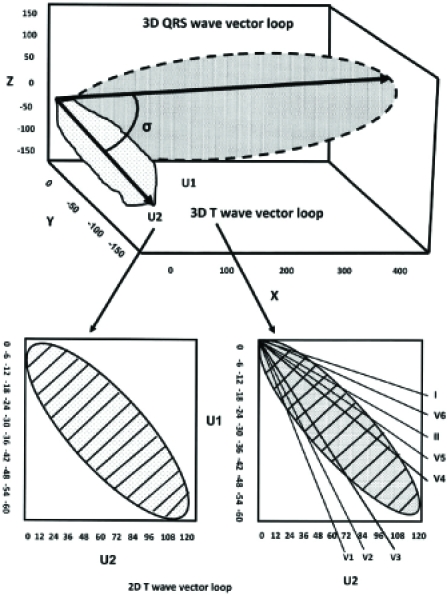
A schematic 3-dimensional view of the QRS and T-wave vector loops. The main vectors of the 2 loops are depicted by arrows and the angle between them is shown (spatial QRS-T angle). Bottom left, the T-wave loop is shown in a 2-dimensional plane with the unipolar axes U1 and U2 and is divided into marked subdivisions. In this plane a hypothetical rectangle encompasses the T-wave loop and is theoretically divided into 100 subdivisions. The T-wave loop dispersion is expressed by the number of the subdivisions that it passes. In this example, the T-wave loop dispersion is 35. The T-wave amplitude is calculated as a fraction of the encompassing rectangle and it is marked by stripes. Bottom right, the reconstruction of the T-wave loop from the vectors of the classical 12-ECG leads. The T-wave morphology dispersion is calculated by the averaging angle between all possible reconstruction vector pairs.

The first report of the measurement of a spatial ventricular gradient appeared in 1954 [9]. The spatial ventricular gradient is actually computed by adding the mean vector representing all of the electrical forces produced by depolarization and the mean vector representing all of the electrical forces produced by repolarization. This is accomplished by forming a parallelogram using the QRS vector and T vector as its sides; the diagonal of the figure is the ventricular gradient (Fig. **1**) [4]. The spatial QRS-T angle measures the vector deviation between the depolarization and repolarization waves by calculating cosine values between the 3-dimensional R- and T-wave loop vectors within the optimized decomposition space. Positive values correspond to large differences in the orientation of the QRS- and T-wave loops (Fig. **1**). The QRS and the T-wave loops assume the same direction as do the QRS and T axes. Consequently, the spatial ventricular gradient points along the long axis of the heart, in the direction of the apex [10]. Furthermore, it should be noted that the vector representing the ventricular gradient points to a direction from a point in the cell in which the duration of the excited state is longest to that in which it is shortest [4]. As the ventricular gradient points to the direction of the briefer action potentials, it can be deduced that corresponding apicobasal and/or endocardial-to-epicardial action potential morphology trends (briefest action potentials apically/epicardially) must necessarily exist. 

**Fig. (2):**
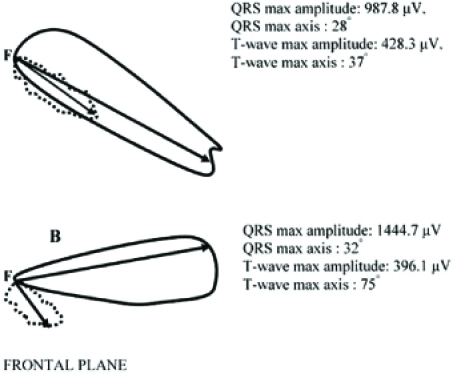
The vectorcardiograms of a healthy subject (A) and of a subject with type 2 diabetes mellitus and hypertension (B) in the frontal plane. The continuous line depicts the loop of the QRS-complex and the dashed line the loop of the T-wave in the frontal plane. On the right corner the max values of the QRS and T-wave vector loops amplitude are shown (calculated in μV). Moreover, the max values of the QRS and T-wave axis in the frontal plane are shown (calculated in degrees). The spatial QRS-T angle (the angle between the two arrows) in the case (A) is 1.55 degrees and in the case (B) is 57.31 degrees.

The frontal plane direction of the normal spatial ventricular gradient is defined as located between +20º and +70º. Its normal direction is described almost parallel with the mean anatomic axis of the ventricles. Accordingly, the range of the normal spatial ventricular gradient is 0º to +90º (Fig. **2**). Although it is more difficult and less accurate to determine the anteroposterior direction of the normal spatial ventricular gradient of the normal adult subject, it is usually located about 10º to 20º anteriorly or posteriorly to the frontal plane [9]. 

The analytical proof that the vectorial QRST integral, referred to as the “spatial ventricular gradient”, is proportional to the volume integral of the action potential duration gradient over the heart was published in 1957 [11]. This analysis established the spatial ventricular gradient as an index for action potential heterogeneity throughout the heart. 

## MECHANISM FOR THE PRODUCTION OF A NORMAL SPATIAL VENTRICULAR GRADIENT

The depolarization process of the ventricles begins at the endocardium and is directed toward the epicardium, creating an electrical force that is directed in the same direction. The repolarization process of the ventricles begins in the ventricular epicardium and is directed toward the endocardium producing an electrical force that is directed in the opposite direction. The QRS complex, which is created by depolarization, develops early in ventricular systole when the pressure in the ventricles is just developing, whereas the T-wave is produced later in ventricular systole after the right and left ventricular peak pressure has passed, but when the intraventricular pressure is much higher than it was when the QRS complex was created [6]. The pressure in the endocardium is higher than in the epicardium and this delays the restoration of electrical charges in the endocardial area of the heart. This phenomenon is thought as a transmyocardial pressure gradient [6]. Therefore, the repolarization process is forced to begin in the epicardium rather than the endocardium. This reveals that the normal adult human heart creates a normal ventricular gradient each time it contracts. The spatial ventricular gradient is directed away from the area of the heart where there is a delay in the repolarization process. 

However, the question of why there is a spatial ventricular gradient in a normal heart remains unanswered by the foregoing mechanism. A possible answer to this might lie in the fact that the ventricles are not homogenous blocks of myocardium, cell types from the extracellular matrix, vascular and nervous tissue co-exist with the myocardial myocytes in a complex 3-dimensional structure [12]. The concept that the transmyocardial pressure gradient is not uniform throughout the ventricular myocardium but is higher in the subendocardium of both ventricles than in the subepicardium is now well founded; the unequal distribution of the endocardial pressure throughout the ventricles delays the time the endocardium begins to repolarize and the duration of the excited state is prolonged. Thus, the mean spatial repolarization vector (T-wave vector) is not directed in exactly the same direction as the mean spatial QRS vector and a spatial ventricular gradient of 0º–60º is present in most normal adult human hearts [6]. Moreover, the aforesaid structural and functional myocardial changes induce aberrations in ionic channel functions and regionally heterogeneous shortening of APDs. In contrast to atrial electrical remodeling, which is mainly related to calcium channels and induced by changes in cycle length, ventricular electrical remodeling might be related mainly to certain potassium channels and induced by changes in cycle length and /or activation sequence. The expression of 8 mRNA for K+ channel is greater in the epicardium than the endocardium and the current density of the channel in respect is likewise greater in epicardium. As a consequence of these greater repolarising currents, the APD is shorter in epicardium [12]. Besides, the widening of the spatial QRS-T angle is frequently associated with an anterior shift of the T axis, suggesting preferential action potential shortening in the anterior wall (epicardial) regions [6]. 

The effect of sustained inhibition of individual K+ currents on various other cardiac ionic currents was recently assessed [13]. The pharmacological induced shortening of the APD and the increased repolarization reserve were accompanied by increased slow delayed-rectifier K+ channel density whereas late Na+ current remained unchanged. This suggested that sustained reductions in individual K+ currents may lead to compensatory upregulation and may also influence repolarization reserve [14]. 

## MECHANISMS RESPONSIBLE FOR AN ABNORMAL SPATIAL VENTRICULAR GRADIENT

As already stated, the QRST integral (total area under the curve over the QT interval) in an ECG lead depends solely on the heterogeneity of the action potential durations in the muscle fiber or otherwise stated the local variations in the excitatory process and not on the order in which the cardiac muscle cells are activated [1, 15]. In other words, the spatial ventricular gradient integrates spatial gradients in the action potential morphology throughout the heart, one major aspect being APD. This was experimentally affirmed in an isolated muscle strip [16] and later on in the complete heart [17]. Recently, multiple studies have searched for the existence of transmural, apicobasal and left ventricular–right ventricular APD gradients [18]. 

During pathophysiological changes (i.e. ischemia, fibrosis or infarction) APD spatial gradients in the heart change dynamically due to dynamic APD changes in the affected area. During a brief initial period, APD spatial gradients are initially prolonged [19] and thereafter shortened [20, 21]. Shortening is more pronounced in the affected epicardial areas than in the endocardial areas [22] and action potential morphology heterogeneity is increased by heterogeneous loss of APD amplitude and shortening of APD in the ventricular epicardium [23]. Moreover, sustained stretches may shorten the APD and flatten the electrical restitution curve, whereas stretches applied at the wavefront prolong the APD. We therefore conclude that when the spatial ventricular gradient is abnormally directed it is likely to be due to localized nonphysiologic causes such as ischemia, fibrosis, or some other pathologic reason. The abnormally directed spatial ventricular gradient may occasionally be directed away from the centroid of the epicardial damage produced by severe generalized epicardial ischemia or severe generalized epicardial damage due to pericarditis [6]. 

Altered ventricular activation sequences cause secondary T-wave changes but do not alter the action potential morphology distribution. To such changes the ventricular gradient should remain insensitive. However, it has been observed that secondary T-wave changes appear to affect the ventricular gradient [24]. This has been ascribed to measurement errors, physiologic variability due to respiration [1], altered electrotonic influences [25], altered cellular electrophysiologic properties due to premature excitation and cardiac memory [26]. 

## THE CLINICAL USE OF THE SPATIAL VENTRICULAR GRADIENT

Dispersion of repolarization of a given heartbeat, electrocardiographically reflected in its T-wave, arises from superimposition of (1) the heterogeneity, throughout the ventricles, of the action potential morphologies for that given heartbeat; and (2) the heterogeneity of the ventricular depolarization instants as they result from the conducted impulse that gave rise to the beat under consideration. Theoretically, a pure primary or secondary T-wave would result when all ventricular myocardium was excited at the same time instant or when all ventricular action potentials had identical shapes, respectively. A hypothetical T-wave that depends only on action potential morphology heterogeneity and not on the depolarization sequence is called “the primary T-wave”, whereas a T-wave that arises on the basis of depolarization heterogeneity only, in the absence of any action potential morphology heterogeneity is called “the secondary T-wave”. The area under the primary T-wave equals the ventricular gradient [27]. In practice, both primary and secondary factors contribute to the T-wave, and these contributions cannot be unravelled by T-wave analysis alone. 

Since its introduction the spatial ventricular gradient has been recognized as a potential ECG tool for discriminating between primary and secondary T-wave phenomena [28]. The ability to assess heterogeneity of the ventricular action potential morphology independent of secondary factors is the power of the ventricular gradient. Although reentrant spiral-based tachyarrhythmia can be initiated in conditions of homogeneous action potential morphology [29], multiple tachyarrhythmias potentially deteriorating into ventricular fibrillation appear in a situation of increased heterogeneity of action potential morphology [30]. Moreover, reducing action potential morphology heterogeneity has an antiarrhythmic effect. Therefore, measurement of the ventricular gradient may considerably contribute to experimental and clinical arrhythmology [31]. 

## INTRODUCTION OF THE SPATIAL QRS-T ANGLE AND ITS RELATIONSHIP TO SPATIAL VENTRICULAR GRADIENT

Recently, the mathematical proof that the integral of the heart vector over the QRST interval is proportional to the volume integral of the heterogeneity of APDs was endorsed [32]. Thus, it was demonstrated that the integral of the heart vector during depolarization (spatial QRS integral) depends on the heterogeneity of the activation instants, whereas the integral of the heart vector during repolarization (spatial T-wave integral) is proportional to the dispersion of repolarization (provided QRS and T do not overlap). This analysis establishes the electrocardiographic relationship between primary factors (APD dispersion, measured in the ECG as the ventricular gradient), secondary factors (dispersion in activation instants, measured in the ECG as the QRS integral), and the resulting dispersion of repolarization (measured in the ECG as the T-wave integral) [33]. Noteworthy, until then, in electrocardiography, dispersion in ventricular activation currently was uniquely assessed by QRS duration, thus neglecting QRS amplitude. However, to electrocardiographically assess the repolarization process, both T-wave amplitude and T-wave area (mostly scalar rather than vectorial) are being used as indices of dispersion of repolarization [34]. 

A situation with increased QRS and T integrals is not necessarily associated with a large ventricular gradient. In a normal heart, pure secondary changes (e.g., altered intraventricular conduction sequence with ventricular ectopy) yield wide and bizarre QRS complexes and T-waves with large QRS and T integrals. However, in this case the angle between the QRS and T axes will be large (the ECG will be discordant), and the ventricular gradient, which is the vectorial sum of the QRS and T integrals, remains unchanged (no primary changes). 

The spatial QRS-T angle has proven to be an important prognostic ECG index [35], can be measured easily, is not affected by observation biases and is likely to be less susceptible to noise and problems of definition than conventional ECG measures of the dispersion of the repolarization duration. 

Although, spatial QRS-T angle and spatial ventricular gradient differ substantially in their methods of calculation [36], they share the same physiological background. They both quantify the deviation between the directions of the ventricular depolarization and repolarization and represent a global measure of the variations of ventricular APD and morphology and they serve as ECG indexes of vulnerability to arrhythmia [36, 37]. However, possibly, combining the spatial QRS-T angle with the ventricular gradient could still further increase its prognostic value, as this would add information about the absence or presence of primary changes. With pure secondary changes, only the spatial QRS-T angle would be enlarged, while the ventricular gradient remains unchanged. With additional primary changes (a less favourable condition), the ventricular gradient would be enlarged as well [38]. 

## THE SPATIAL QRS-T ANGLE: EPIDEMIOLOGICAL DATA

Previous studies have concluded that obesity increases the spatial QRS-T angle independent of positional changes and that this effect is in the direction of relative left ventricular ischemia [39, 40]. The question whether a larger spatial QRS-T angle might not represent, in fact, a slight degree of ischemia since this is compatible with the higher incidence of degenerative heart disease in obese subjects was aroused [40]. Moreover, spatial QRS-T angle was found to be significantly associated with height and was larger in shorter than in taller subjects. This association was explained by a higher ratio of heart volume/chest volume in shorter men. The influence of height was attributed to extracardiac rather than to cardiac variables [41]. In the same study there also was a highly significant decrease of the spatial QRS-T angle from horizontal to vertical position and the interindividual variability of spatial QRS-T angle was found to be smallest in subjects with vertical and semivertical hearts [40]. 

A correlation between the mean QRS and T vectors dependent on the spatial QRS-T angle between these vectors was also demonstrated and showed the superiority of spatial vector analysis over conventional electrocardiographic analysis [42]. In the analysis of scalar ECGs, the error of projection of spatial vectors on any given lead, and the inaccessibility of spatial QRS-T angle, had obscured the relationship between QRS complex and T wave. By means of spatial vector analysis, therefore, it was possible to decide a controversial issue in the electrocardiographic literature on a fairly safe basis. Therefore it was concluded that a high spatial QRS-T angle, even within physiologic limits, is of importance since it tends to abolish the physiologic correlations between QRS and T. 

Since heterogeneity of ventricular repolarization is linked to arrhythmogenesis and gender differences in the incidence of torsade de pointes and sudden cardiac death are often present, the repolarization homogeneity and its circadian pattern in men and women was investigated in another study. In healthy subjects 24-hour 12-lead digital ECGs were repeatedly recorded during the first day, as well as one week and one month after the first record. Lower spatial QRS-T angle values corresponded to low repolarization heterogeneity and over the entire 24 hours women showed significantly lower spatial QRS-T angle values than men. There was no significant gender difference in the extent of the circadian pattern. Both males and females showed lower spatial QRS-T angle values (greatest homogeneity of repolarization) during the night and a steep increase in heterogeneity in the morning [43]. The only gender difference was that in women at all heart rates the sequence of repolarization replicated more closely the sequence of depolarization and the spatial QRS-T angle had greater values over the entire range of investigated RR intervals (and thus the “global” repolarization heterogeneity less pronounced), whereas in men localized repolarization was more heterogeneous especially at fast heart rates. This suggests that both global and regional repolarization heterogeneity are increased at faster heart rates in both genders [44]. A recent study investigated and documented the reflection of psychoemotional stress in the body surface potential distribution. In young men with no cardiovascular history body surface isointegral maps of cardiac activation and recovery at rest and during the test of mental arithmetic were recorded. The results showed a significant increase in QRS-T angle values over the sternum and right precordium during mental stress which contributed to analogically localized increments also in the spatial QRS-T angle. In conclusion, the spatial QRS-T angle was strong enough to distinguish the stress-induced changes in the superficial cardiac electric field. These adrenergic transient alterations in ventricular recovery may be of importance in subjects at risk for ventricular arrhythmias [45]. 

Patients suffering from panic disorder present an increased risk of myocardial ischemic changes. Using classical ECG methods, this risk cannot be evaluated in most patients. The spatial QRS-T angle was measured in a study of panic disorder patients without any seizures and pharmacological treatment and without cardiovascular symptoms and was found to be increased compared with the control group. This difference persisted even in the period free of a panic attack and was attributed to activation of the adrenergic system, blocking of cholinergic innervation and loss of autonomic nervous system control. The results of this study showed that changes in the heart electric field parameters occurred in panic disorder patients when compared to the control group [46]. 

The impact of the circulatory effects of cigarette smoking on spatial QRS-T angle has also been evaluated in a population of young, healthy, male subjects. The spatial QRS-T angle was higher in smokers compared to non-smokers, whereas the QT dispersion did not differ between the two groups. The differences in the heterogeneity of ventricular repolarization between smokers and non-smokers were mainly attributed to heart rate differences between the two studied groups [47]. 

It is widely accepted that n-3 fatty acids intake may reduce the risk of sudden death by preventing life-threatening cardiac arrhythmia [48]. The effect of n-3 fatty acids on the spatial QRS-T angle in apparently healthy men and women was recently investigated in order to detect clues to the mechanism by which n-3 fatty acids affect the electrophysiology of the heart. The study subjects received either 1.5 g n-3 fatty acids daily or placebo for 12 weeks and ECGs were recorded before and after intervention. The spatial QRS-T angle was not affected by n-3 fatty acids supplementation; however, an effect of these fatty acids on spatial QRS-T angle in more susceptible populations can not be excluded [49]. 

Recently, the spatial QRS-T angle was also found to be strongly and independently associated with the K897T polymorphism of the KCNH2 (HERG) gene coding for the rapidly activating delayed rectifier K+ channel. This polymorphism influences cardiac repolarization and its functional significance for cardiac electrical properties was for the first time assessed by spatial QRS-T angle in a population consisting of healthy middle-aged subjects. Subjects with a less common genotype had smaller spatial QRS-T angle values which reflects desynchronization between depolarization and repolarization and is associated with an increased risk of cardiac mortality [50]. 

## THE SPATIAL QRS-T ANGLE ASSESSES VENTRICULAR REPOLARIZATION ALTERATIONS IN SUBJECTS WITH EARLY REPOLARIZATION

The ECG features of early repolarization (ER) in apparently healthy subjects have been studied extensively [51] and many previous studies evaluated the morphological characteristics of this ECG variant and its differences from diseases of abnormal ventricular repolarization [52, 53]. However, a recent study conducted the first systematic quantification of ventricular repolarization in subjects with ER by evaluating ECG and spatial vectorcardiographic (VCG) descriptors of ventricular repolarization in subjects with ER [54]. Besides, the studied indices were associated with the respective indices of ventricular depolarization. The spatial QRS–T angle was significantly higher in ER subjects than in controls indicating that the repolarization vectors are more shifted away from the depolarization vectors in ER subjects than in age-matched healthy controls. This was attributed to the inhomogeneous repolarization of the left ventricle in ER subjects [55]. There was no significant association between the VCG and/or ECG descriptors of ventricular depolarization or repolarization in ER subjects and this may be explained in part by the presence of inhomogeneous autonomic tone influence on the heart in these subjects [56] and the different quality of information on cardiac electrophysiology offered by the respective studied indices [57]. 

## THE SPATIAL QRS-T ANGLE AS A MARKER OF THE REPOLARIZATION HETEROGENEITY IN ARRHYTHMIA

Ibutilide is a pure class III antiarrhythmic agent that acts predominantly by prolonging myocardial action potential duration and is considered to be a useful agent for pharmacologic cardioversion of recent-onset atrial fibrillation or flutter [58]. Ibutilide is used for the pharmacologic cardioversion of atrial fibrillation or flutter and its effect on QT interval prolongation has been demonstrated previously [59]. The effects of ibutilide on ECG and VCG descriptors of ventricular repolarization were recently studied by repeated infusions of ibutilde in a group of consecutively recruited patients with atrial fibrillation or flutter of recent onset [60]. The ECG (QT interval, rate-corrected QT interval and QT dispersion) and the spatial VCG descriptors (spatial T amplitude and spatial QRS-T angle) were calculated before (baseline ECG) and 30 min after the start of ibutilide infusion (postinfusion ECG). In the group of patients who were cardioverted to sinus rhythm after ibutilde perfusion, the ECG markers of ventricular repolarization were significantly increased from baseline to the postinfusion ECG, whereas the spatial T amplitude and the spatial QRS-T angle did not differ between those two ECGs. In the group of patients in which atrial fibrillation or flutter persisted spatial T amplitude was significantly increased and spatial QRS-T angle was significantly decreased post infusion compared with baseline ECG. In conclusion, while temporal measures of ventricular repolarization were significantly affected in all patients who received ibutilide infusion for atrial fibrillation or flutter cardioversion, spatial VCG descriptors of ventricular repolarization were significantly altered only in those patients who fail to respond to the drug, implying a dose-related effect of ibutilide on the different aspects of ventricular repolarization. However, more and larger studies are needed to assess the ability of spatial QRS-T angle to predict the occurrence of ibutilide-induced proarrhythmia. 

## THE SPATIAL QRS-T ANGLE AS A MARKER OF VENTRICULAR REPOLARIZATION IN ARTERIAL HYPERTENSION AND LEFT VENTRICULAR HYPERTROPHY

It is widely recognised that systemic hypertension affects up to 25% of the adult population and is a potent cardiovascular risk factor [61]. Left ventricular hypertrophy (LVH) in hypertensive patients results in inhomogeneity of ventricular repolarization, favouring the propensity to ventricular tachyarrhythmias [62], and the occurrence of electrophysiological changes in response to ventricular pressure or volume overload has been well documented [63]. Although the factors predisposing to electrical instability and arrhythmic death are not well established [64], it has been reported that myocardial hypertrophy alters the ionic channels that are operative during the early repolarization phase [65, 66]. Furthermore, LVH is characterised by an increase in collagen interstitial matrix that may also lead to alterations in ventricular repolarization [67] by augmenting transmural repolarization gradients and by cellular uncoupling [68]. In addition, repolarization gradients in the transmural axis generate the T-wave on the ECG [69]. Thus, it is possible that T-wave morphology contains important information on both ventricular repolarization and arrhythmic vulnerability in patients with LV hypertrophy. 

The variability in the QT interval duration among the different leads of a surface 12-lead ECG (QT dispersion) is supposed to reflect local differences in the recovery time of the myocardium [70]. Although, high QT dispersion values have been reported in patients with systemic hypertension [71], and that QT dispersion values decrease when adequate blood pressure (BP) control is achieved [72], well-known difficulties in the ability to measure the QT interval in all leads contribute to poor reproducibility of QT dispersion. Besides, the rather weak correlation between QT dispersion and BP levels found in previous studies reduces its power to assess arrhythmia risk prospectively. 

Previous studies have reported a widened spatial QRS-T angle in patients with eccentric LVH [73]. The spatial ventricular gradient and the spatial QRS-T angle were examined in a group of patients with angiographically determined eccentric LVH, as compared with matched control subjects. The spatial ventricular gradient in the eccentric LVH group increased significantly in comparison with the control group. Furthermore, widening of the spatial QRS-T angle was observed only in the subjects with LVH. In cases of mild or moderate LVH, normal spatial ventricular gradient and normal spatial QRS-T angle were observed. It was thus concluded that the magnitude of the spatial ventricular gradient increases proportionally to an increase in total left ventricular muscle volume in hypertrophy and that the widening of the spatial QRS-T angle observed in LVH is due mainly to an alteration in the ventricular gradient [74]. A decrease in the frontal or the horizontal counterparts of the spatial QRS-T angle in patients following treatment of hypertension has also been reported [75]. A widened QRS-T angle in patients with LVH was also reported when Grant’s vectorial method was used [76]. 

Recently, the ability of spatial QRS-T angle to discriminate between hypertensives with high or low BP was demonstrated in treated hypertensives who were classified in a high (systolic BP > 160 mm Hg or diastolic BP > 95 mm Hg), or a low (systolic BP < 160 mm Hg and diastolic BP < 95 mm Hg) BP group. The spatial QRS-T angle was higher in patients with high compared to those with low BP, whereas all conventional ECG markers of the dispersion of ventricular repolarization duration failed to demonstrate significant differences between hypertensives with high or low BP. The spatial QRS-T angle was significantly increased in those treated hypertensive patients who showed repeatedly high BP values, therefore it may be suggested that the angle between the directions of ventricular depolarisation and repolarization is a sensitive marker of the repolarization alterations in systemic hypertension [77]. The ability of the spatial QRS-T angle to distinguish hypertensives with low from those with high BP was not attributed to differences in the prevalence rates of LVH between hypertensives with low and those with high BP, since the spatial QRS-T angle was able to distinguish the patients who did not fulfil the criteria for LVH according to their BP levels. 

In patients without overt coronary artery disease (CAD), T-wave morphology descriptors assessing spatial repolarization variation were evaluated and were found to be altered with echocardiographic LVH, and also to have a significant correlation to LV mass. The increased spatial variation in T-wave morphology in patients with LVH seems to result from a significant relationship between the spatial vectorcardiographic parameters and the LV wall thickness [78]. 

Sudden cardiac death is often the first manifestation of CAD even in mildly symptomatic patients with hypertension and mild to moderate heart failure. Epidemiological studies show that LVH and hypertension in CAD increases the risk for cardiovascular events including sudden cardiac death [79]. Three-dimensional vectorcardiographic monitoring was performed during coronary angioplasty in a population of patients with CAD. Data collection was performed at rest and at the time of maximum ischemia during coronary occlusion. CAD patients with LVH had the most abnormal baseline repolarization and a significantly more pronounced repolarization response during coronary occlusion, as evaluated by wider spatial QRS-T angle values. In multiple linear regression analysis the width of the QRS-T angle was increased in the presence of LVH, hypertension, and previous myocardial infarction. The presence of LVH was the most important factor in the analysis, while the number of the diseased vessels only had minor influence on the response to ischemia [80]. 

## THE SPATIAL QRS-T ANGLE AS A MARKER OF REPOLARIZATION HETEROGENEITY IN MYOCARDIAL INFARCTION AND CORONARY ARTERY DISEASE

An infarcted area is electrically inert and distorts the normal spread of excitation. The net effect is that the electrical forces influenced by the “dead zone,” are directed away from the area of myocardial infarction. Therefore, the spatial characteristics of the T loop morphology are altered in the infarcted myocardium [81]. Although the QT dispersion has been used to quantify the dispersion of ventricular refractoriness and has exhibited significant dynamic changes following the natural history of the infarction [82] its poor reproducibility reduces its power to assess the risk of arrhythmia prospectively [70]. The presence of a direct link between the heterogeneity of ventricular repolarization and QT dispersion has been challenged [57] and the differences in QT interval duration have been attributed to the different projections of the spatial T-wave loop into individual ECG leads, rather than to regional heterogeneity of myocardial repolarization. 

The spatial QRS-T angle as a non-dipolar content of the T-wave and a measure of the local repolarization inhomogeneity was studied in a population of patients with ST-elevation myocardial infarction (STEMI) treated with a thrombolytic agent, during the initial course of STEMI when local repolarization inhomogeneity is markedly increased. The study concluded that patients with thrombolyzed STEMI have increased values of spatial QRS-T angle and non-dipolar content of the T-wave. Resolution of the ST-elevation was associated with a decrease and the increased non-dipolar content reflected a property of the repolarization phase, which is related to but separated from the ST-elevation [83]. 

The ability of the spatial QRS-T angle to quantify ventricular repolarization in order to discriminate among different subsets of postinfarction patients was evaluated in a group of consecutively recruited patients with acute myocardial infarction (MI) [84]. The ECG markers (QT interval, QT dispersion) and the VCG markers were manually measured from digitally recorded ECGs. The spatial T amplitude and the spatial QRS-T angle did not differ between patients with recent and those with old (> 6 months) MI. QT dispersion was significantly lower in patients with an old MI than in patients with a recent one. The spatial repolarization descriptors showed better short-term reproducibility than the dispersion indices. In conclusion, the spatial T amplitude and the spatial QRS-T angle are accurate measures of ventricular repolarization that do not differ between patients with recent and those with old MI. However, future studies should clarify the ability of the spatial T loop morphology analysis to offer a more precise and reproducible measure of ventricular repolarization. Furthermore, the ability of these spatial repolarization descriptors to identify high risk patients for ventricular arrhythmias should be prospectively assessed. 

The effects of thrombolysis on vectorcardiographic descriptors of ventricular repolarization were studied in 70 consecutively recruited patients with acute MI [85]. The study population underwent digital 12-lead ECG before and at 3 hours after thrombolysis. Angiography revealed potency of the infarct-related coronary artery after thrombolysis in 52 (74%) patients (Group A) and occlusion in 18 (26%) (Group B). The spatial T amplitude was decreased significantly more in Group A than in Group B patients after thrombolysis, while the spatial QRS-T angle was decreased in Group A but increased in Group B patients. In conclusion, both vectorcardiographic descriptors were significantly affected by thrombolysis in patients with acute MI and thus can be constituted as potential markers of thrombolysis efficacy. Both vectorcardiographic markers and especially spatial QRS-T angle provide global measures of ventricular repolarization that could be used for the prediction of patients’ outcome [37]. Future studies are needed to assess the power of these spatial repolarization descriptors to offer a more precise and reproducible measure of ventricular repolarization and to predict arrhythmic events and cardiac mortality in postinfarction patients in a prospective way. 

Furthermore, perioperative MI is a known major complication after coronary artery bypass graft surgery. However, the reliability of the classic ECG for the diagnosis of perioperative MI in cardiac surgery has often been questioned and a large percent of the patients present diagnostic difficulties due to unspecific elevation of enzymes and uncertainties regarding ECG changes in the postoperative course [86]. Since vectorcardiography is considered superior to ECG for the diagnosis of MI, the usefulness of the spatial vectorcardiographic changes for diagnosis of perioperative MI were studied in a population of patients undergoing coronary surgery. The spatial vectorcardiographic parameters were calculated before and after surgery. Better than the Q-waves on classic ECG, the spatial QRS-T angle and its components were related to sustained elevation of plasma troponin-T and of the other biochemical markers of myocardial injury and also to impaired clinical course. The results of this study confirmed that vectorcardiography is superior to classic ECG in detection of myocardial injury in coronary surgery [87]. 

The usefulness of the spatial QRS-T angle variability for detecting the presence of ischemia was evaluated in a group of patients with CAD and in patients with chest pain and no CAD in comparison with healthy controls. The values of the spatial QRS-T angle were significantly higher in patients with CAD than in the other two groups. The coefficient of variation of the values of the spatial QRS-T angle was the most sensitive (82%) and specific (91%) marker for the diagnosis of CAD. This study suggested that the variability of the values of the spatial QRS-T angle is a reliable indicator of CAD [88]. 

## THE INDEPENDENT PROGNOSTIC VALUE OF SPATIAL QRS-T ANGLE

The widening of the spatial QRS-T angle in subjects without clinical evidence of CAD can be considered as a subclinical abnormality and reflects an abnormal sequence of ventricular repolarization. As already stated, the mechanism of the widening of the spatial QRS-T angle is inherently associated with structural and functional myocardial changes inducing aberrations in ionic channel functions and regionally heterogeneous shortening of action potential durations. When the ventricular excitation sequence is relatively normal, the widening of the spatial QRS-T angle commonly reflects an anterior shift of the T-wave, suggesting preferential action potential shortening in the epicardial regions [89]. 

The prospective population-based Rotterdam Study was the first to assess the prognostic importance of the spatial QRS-T angle for fatal and non-fatal cardiac events in men and women aged 55 years and over. In this study wider spatial QRS-T angles were associated with increased hazard ratios of cardiac death, non-fatal cardiac events, sudden death and total mortality. Noteworthy, none of the classical cardiovascular risk factors and ECG predictors provided larger hazard ratios. Additionally, after adjustment for all risk factors for macrovascular disease, the association between the spatial QRS-T angle with fatal events remained strong [35]. 

Another study assessed and confirmed the predictive value of the spatial QRS-T angle for fatal cardiac events in an elderly general population. Wider spatial QRS-T angle values showed significantly increased hazard ratios for fatal and nonfatal cardiac events even after adjustment for the cardiovascular risk factors and other ECG risk indicators. None of the established cardiovascular and ECG risk factors had larger hazard ratios than the spatial QRS-T angle [90]. 

Population-based studies have shown low or non-significant prognostic value for ECG abnormalities in women [91]. However, recently hazard ratios for ECG variables for combined fatal and nonfatal CAD events and for CAD mortality was evaluated in the Women’s Health Initiative (WHI) Study during a follow-up of up to 9.2 years. Spatial QRS-T angle and ECG-demonstrated myocardial infarction (ECG-MI) were the strongest predictors of CAD events, while in a multi-adjusted risk model the spatial QRS-T angle, ECG-MI, QRS nondipolar voltage and reduced heart rate variability were the dominant predictors of CAD mortality. It must be noted that QT interval prolongation and prolongation of its dispersion were not among the dominant predictors of CAD events and mortality [92]. This is in accordance with previous studies which reported conflicted results on the association between QT prolongation and CAD mortality in women [91, 93]. This important study concluded that ventricular repolarization abnormalities in postmenopausal women are as important predictors of CAD events and CAD mortality as ECG-MI and other QRS abnormalities and warrant attention in future investigations [92]. 

Having in consideration that the information is limited about ECG predictors of the risk of incident congestive heart failure (CHF), particularly in women without overt manifestations of cardiovascular disease (CVD), the same study evaluated hazard ratios for incident CHF and all-cause mortality during a 9-year follow-up. Among the ECG parameters studied, the spatial QRS-T angle was the dominant ECG predictor of all-cause mortality and had a significant interaction with CVD status requiring stratification [94]. 

Widening of the spatial QRS-T angle has also been reported and associated with excess CAD mortality risk in a previous study [95], and another investigation reported increased mortality risk for abnormal T axis deviation [96], related to a widening of the spatial QRS-T angle. The heterogeneity of myocardial repolarization, assessed by T-wave analysis in the ECG, was found to confer long-term independent prognostic information in a large population of males with known CVD. Similar predictors of survival were age, presence of LVH, and left ventricular ejection fraction [97]. Deceased patients had significantly higher heterogeneity of repolarization as compared with patients alive at the end of a follow-up period of > 10 years and the spatial QRS-T angle was independently predictive of cardiovascular events. 

The predictive value of the spatial ventricular gradient in the prognosis of MI was examined in another study in survivors of acute Ml. During a follow-up of 5 years, higher spatial QRS-T angle values were independent predictors of the 1-year and 5-years cardiac mortality, together with low (< 33%) ejection fraction for the 5-year mortality and low heart rate variability for the 1-year cardiac mortality; this was valid for both genders. The authors concluded that the spatial QRS-T angle is an independent predictor of cardiac death in survivors of acute MI [98]. 

In the strategies for management of antiretroviral therapy trial, the effect of different strategies of continuous antiretroviral therapy on the incidence of ECG abnormalities was examined in 5472 patients with AIDS. Incidence of specific ECG abnormalities during a mean follow-up of 16 months and changes in measures of cardiac function were compared between study arms for the participants who had been followed-up adequately. Increased spatial QRS-T angle values at baseline predicted independently the incidence of cardiovascular disease events regardless of antiretroviral medications and clinical measures of disease severity [99]. 

## CONCLUSIONS AND HOPES FOR THE FUTURE

In the just over 100 years since the first ECG was performed, the ECG has become the most extensively used noninvasive diagnostic and prognostic tool in cardiology and has impressive, if imperfect, utility for rhythm analysis, detection of ischemic and hypertrophic heart disease, and outcome prediction in a variety of clinical settings, with a large body of literature that illustrates and supports these applications [100]. 

However, it is becoming increasingly recognized that physicians tend to underestimate the risk of adverse cardiovascular events [101]. Although a close link between an increased heterogeneity of ventricular repolarization and arrhythmogenicity has been demonstrated in many previous as well as very recent experimental studies [102, 103], repolarization abnormalities are often ignored as inconsequential. QT prolongation is well known to be associated with arrhythmia provocation; however the predictive value of its moderate prolongation and the underlying mechanisms for arrhythmogenic risk in asymptomatic subjects is less clear. In many studies QT prolongation has been found to have a nonsignificant association with mortality when evaluated specifically in subjects without CAD [104, 105]. 

The results from recent major population-based studies suggest that in subjects with and without prior CVD, widening of the spatial QRS-T angle indicates an abnormal sequence of ventricular repolarization and is a dominant ECG predictor of future CAD events and CAD death. Spatial QRS-T angle is currently not routinely reported in clinical electrocardiography; however its value could prove a fertile area for future research. 

Measurement of the spatial QRS-T angle is likely to be less susceptible to noise and problems of definition than many of the more conventional ECG parameters. Accurate determination of waveform recognition points, in particular the end of the T wave, is less critical for calculation of the QRS-T angle. Thus, the spatial QRS-T angle is likely to be a much more robust and reproducible measurement than QT dispersion, which has also been used to quantify ventricular repolarization but was shown to have several methodological limitations. 

In light of the low cost, the widespread availability of the ECG and the increasing economic burden of the health-related problems, it is imperative to establish new, low-cost markers for risk stratification and prevention strategies. Assessment of the spatial QRS-T angle warrants serious consideration for intensified primary and secondary prevention efforts and can easily be included in the diagnostic quiver of the clinicians in their everyday clinical practice.
