# Erratum

**DOI:** 10.2174/157340310793566091

**Published:** 2010-11

This is with reference to the article entitled, “**Assessment of the Spatial QRS-T Angle by Vectorcardiography: Current Data and Perspectives**”, by Christina Voulgari and Nicholas Tentolouris published in *Current Cardiology Reviews* Journal, 251-62 November **2009**, Vol. *5*, No. *4*, pp. 251-62.

The authors have overlooked to cite the paper entitled, “**Elucidation of the spatial ventricular gradient and its link with dispersion of repolarization**”, by Draisma HH, Schalij MJ, van der Wall EE, Swenne CA published in *Heart Rhythm* Journal, 2006, Vol. 3, No. *9*, pp. 1092-9 in the above mentioned article.

